# College and Hospital Notes

**Published:** 1905-02

**Authors:** 


### COLLEGE AND HOSPITAL NOTES.

THE Manhattan Eye, Ear and Throat Hospital, New York, will erect a new building on 63d street, to be twelve stories high and to accommodate about 400 ward patients and 50 private patients. A feature of the Manhattan hospital is its postgraduate department. Here any physician holding a recognised degree may take a three months' course in various phases of surgery connected with the special work of the hospital.

THE Batavia Hospital is to receive a gift of $5,000 from Mrs. Adelaide R. Kenney, as indicated by the following letter addressed to the board of directors:

"Inasmuch as the town is endeavoring to secure three new factories, which will be of great benefit to Batavia, but will necessitate an expenditure by the village, and as we need the hospital enlarged, and as we do not wish to ask the people for more money, if the directors will secure satisfactory plans which will include a nurses' home, an operating room, and more wards, to be built for $5,000, I will give the $5,000."

For the generous gift the directors by a rising vote extended to Mrs. Kenney a most hearty vote of thanks.

THE University of Buffalo announces the second series of university lectures to be given by the following named gentlemen: Frank Hyatt Smith, Lewis Stockton, Harlow C. Curtiss and Herbert P. Bissell, each of whom will deliver a course, respectively, on English literature, Popular government, American history, and German literature. The fee for each course is $5.00.

BRIGHAM HALL, a hospital for nervous and mental diseases, located at Canandaigua, N. Y., has recently issued a handsome brochure, beautifully illustrated, giving an account of the institution. It was founded in 1855, and is one of the most famous private hospitals for the treatment of the class of diseases referred to, in this country. It is under the charge of Dr. D. R. Burrell, who ranks among the most eminent alienists of the present day.

FOUR of the trained nurses who have recently returned from Japan, where they were serving in the base hospital at Hiroshima under the direction of Dr. Anita Newcomb McGee, have volunteered to go to Panama to nurse in the hospitals of the Canal Commission.—*Medical Age.*

